# Neurotoxic/Neuroprotective Effects of Clozapine and the Positive Allosteric Modulator of mGluR2 JNJ-46356479 in Human Neuroblastoma Cell Cultures

**DOI:** 10.3390/ijms24032054

**Published:** 2023-01-20

**Authors:** Patricia Gassó, Albert Martínez-Pinteño, Natalia Rodríguez, Santiago Madero, Marta Gómez, Alex G. Segura, Clemente García-Rizo, Constanza Morén, Sergi Mas, Eduard Parellada

**Affiliations:** 1 Department of Basic Clinical Practice, University of Barcelona, 08036 Barcelona, Spain; 2 Institut d’Investigacions Biomèdiques August Pi i Sunyer (IDIBAPS), 08036 Barcelona, Spain; 3 Centro de Investigación Biomédica en Red de Salud Mental (CIBERSAM), 28029 Madrid, Spain; 4 Barcelona Clínic Schizophrenia Unit (BCSU), Department of Psychiatry, Institute of Neuroscience, Hospital Clínic of Barcelona, University of Barcelona, 08036 Barcelona, Spain; 5 Centro de Investigación Biomédica en Red de Enfermedades Raras (CIBERER), 28029 Madrid, Spain

**Keywords:** JNJ-46356479, positive allosteric modulator, mGluR2, apoptosis, antipsychotic, schizophrenia

## Abstract

Current antipsychotics (APs) effectively control positive psychotic symptoms, mainly by blocking dopamine (DA) D2 receptors, but have little effect on negative and cognitive symptoms. Increased glutamate (GLU) release would trigger neurotoxicity, leading to apoptosis and synaptic pruning, which is involved in the pathophysiology of schizophrenia. New pharmacological strategies are being developed such as positive allosteric modulators (PAMs) of the metabotropic GLU receptor 2 (mGluR2) that inhibit the presynaptic release of GLU. We previously reported that treatment of adult mice with JNJ-46356479 (JNJ), a recently developed mGluR2 PAM, partially improved neuropathological deficits and schizophrenia-like behavior in a postnatal ketamine mouse model. In the present study, we evaluated, for the first time, the putative neuroprotective and antiapoptotic activity of JNJ in a human neuroblastoma cell line and compared it with the effect of clozapine (CLZ) as a clinical AP with the highest efficacy and with apparent utility in managing negative symptoms. Specifically, we measured changes in cell viability, caspase 3 activity and apoptosis, as well as in the expression of key genes involved in survival and cell death, produced by CLZ and JNJ alone and in combination with a high DA or GLU concentration as apoptosis inducers. Our results suggest that JNJ is not neurotoxic and attenuates apoptosis, particularly by decreasing the caspase 3 activation induced by DA and GLU, as well as increasing and decreasing the number of viable and apoptotic cells, respectively, only when cultures were exposed to GLU. Its effects seem to be less neurotoxic and more neuroprotective than those observed with CLZ. Moreover, JNJ partially normalized altered expression levels of glycolytic genes, which could act as a protective factor and be related to its putative neuroprotective effect. More studies are needed to define the mechanisms of action of this GLU modulator and its potential to become a novel therapeutic agent for schizophrenia.

## 1. Introduction

Schizophrenia is a heterogeneous and complex psychiatric disorder, the etiopathogenesis of which remains poorly understood [1]. There are different theories involved in the pathophysiology of this psychiatric disorder. The dopamine (DA) hypothesis is one of the main theories that explains the development of positive psychotic symptoms. In recent decades, this has led to the glutamate (GLU) hypothesis, which also plays a key role in schizophrenia pathophysiology, particularly in the development of negative symptoms and cognitive dysfunction [2,3].

Evidence from pharmacological, postmortem, brain imaging and genetic studies supports the role of glutamatergic dysregulation in schizophrenia [4]. This theory was proposed based on the capacity of N-methyl-D-aspartate (NMDA) GLU receptor antagonists, such as ketamine, to mimic the positive, negative and cognitive symptoms of schizophrenia in healthy individuals and to exacerbate psychotic symptoms and cognitive decline in patients with the disease [5]. The blockade of NMDA receptors in gamma-aminobutyric (GABAergic) interneurons, which express calcium-binding protein parvalbumin, generates a hyperglutamatergic condition as a consequence of decreased inhibitory control of excitatory pyramidal neurons. This leads to increased GLU release and excessive stimulation of glutamatergic receptors [3], resulting in excitotoxic damage and cognitive impairment [6]. Dendritic spine alterations are thought to be related to the pathology of schizophrenia; there are many reports of decreases in dendritic spine density in different brain regions of patients with the disease [7]. An excess of GLU would trigger neurotoxicity, apoptosis and synaptic pruning by local activation of caspase 3, leading to a loss of density of dendritic spines in critical brain areas [8,9]. Previous results reported by our group support this hypothesis. We have observed increased apoptotic susceptibility in primary fibroblast cell cultures from antipsychotic (AP)-naïve patients with a first psychotic episode [10], which showed alterations in the expression of genes involved in the apoptotic pathways [11]. Moreover, correlations between altered apoptotic markers and both the volume of specific brain regions and the concentration of neurometabolites were found by magnetic resonance imaging [12]. The mechanisms of GLU-induced cell damage in neuronal-like cell models may differ from those found in primary neuronal cell cultures. It has been reported that cell exposure to high concentrations of GLU evokes oxidative stress and oxytosis, which could engage various cell death programs such as caspase 3 and/or caspase 3-independent apoptosis or necroptosis [13].

Current pharmacological treatment of schizophrenia is based on the use of both first- and second-generation APs. These block postsynaptic dopamine D2 receptors and can be effective in the control of positive psychotic symptoms but have little effect on the negative and cognitive symptoms [14]. Clozapine (CLZ) is a second-generation AP with low D2 potency but with an established benefit in patients with treatment-resistant schizophrenia due to its superior efficacy among all AP agents and with apparent utility, according to existing evidence, in managing the negative symptoms of schizophrenia [15,16,17]. Given the importance of GLU in the pathophysiology of this disease, new pharmacological strategies are currently being developed to reduce the hyperglutamatergic state in order to improve treatment of negative and cognitive symptoms. One such example is 8-trifluoromethyl-3-cyclopropylmethyl-7-[(4-47(2,4-difluorophenyl)-1-piperazinyl)methyl]-1,2,4-triazolo[4,3-α]pyridine (JNJ-46356479), a new selective and orally bioavailable positive allosteric modulator (PAM) of the metabotropic GLU receptor 2 (mGluR2) that inhibits the presynaptic release of GLU [18,19]. We recently found that treatment of adult mice with JNJ-46356479 (JNJ) partially improves neuropathological deficits and schizophrenia-like behavior in a postnatal ketamine mouse model [20]. Particularly, JNJ treatment partially ameliorates the reduction in neurons expressing parvalbumin in the medial prefrontal cortex and dentate gyrus that is induced by postnatal ketamine exposure. JNJ could play a neuroprotective role through different mechanisms of action, including an antiapoptotic effect that may be more potent than that of other APs we have previously observed in neuroblastoma cell culture [21]. Interestingly, some mGluR2/3 agonists have previously shown neuroprotection against different neurotoxic insults in different cell cultures [22], including neuroblastoma [23,24]. The human neuroblastoma cell line displays neuronal properties and is a popular in vitro model used in neuropsychiatric research including schizophrenia [23,24,25,26] that has been widely used to test pharmacological effects of drugs including APs [26,27,28,29].

In this study, we aimed to evaluate, for the first time, the putative neuroprotective and antiapoptotic activity of JNJ in a human neuroblastoma cell line and compare it with the effect of CLZ as a clinical AP with an apparent efficacy in treating negative symptoms. Specifically, we measured changes in cell viability, caspase 3 activity and apoptosis, as well as in the expression of key genes involved in survival and cell death signaling pathways produced by CLZ and JNJ alone and in combination with a high DA or GLU concentration as apoptosis inducers.

## 2. Results

### 2.1. Effects of Clozapine (CLZ) and JNJ-46356479 (JNJ) Alone and in Combination with Dopamine (DA) or Glutamate (GLU) on the Viability of SK-N-SH Cells

**Figure 1:**
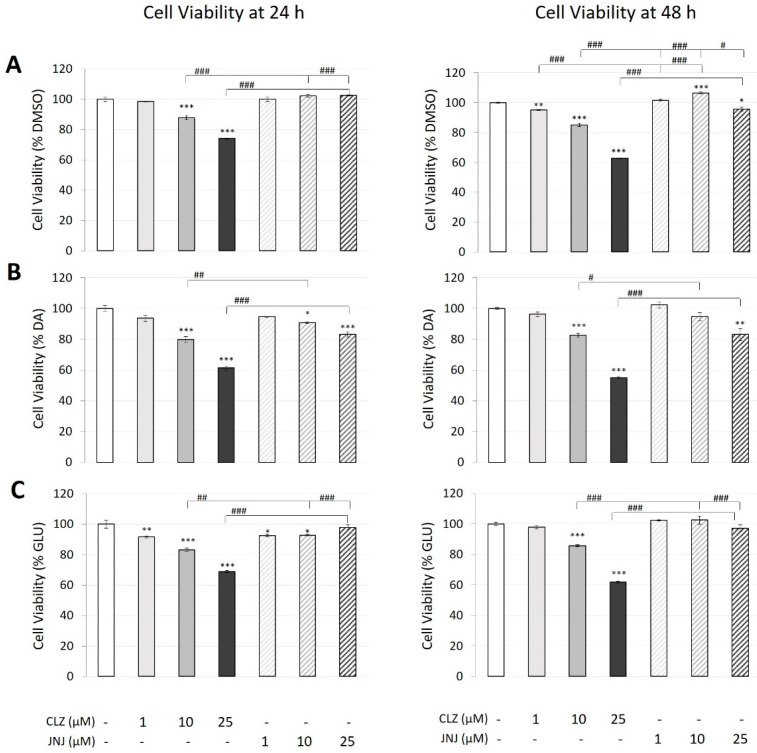
Effects of different concentrations (1, 10 and 25 μM) of clozapine (CLZ) and JNJ-46356479 (JNJ) alone (**A**), in combination with dopamine (DA) at 100 μM (**B**) and in combination with glutamate (GLU) at 80 mM (**C**) on the viability of SK-N-SH cells after 24 h and 48 h as measured by Alamar Blue^®^ assays. The values represent percentages of the control (DMSO-treated), DA-treated or GLU-treated cells and are expressed as means ± S.E.M (n = 3). Two-way ANOVA was performed to assess the drug treatment and concentration effect, and Bonferroni’s post hoc test was used for pair comparisons. * *p* ≤ 0.05, ** *p* ≤ 0.01, *** *p* ≤ 0.001 vs. control. ^#^ *p* ≤ 0.05, ^##^ *p* ≤ 0.01, ^###^ *p* ≤ 0.001 included in square brackets indicate comparisons between CLZ and JNJ.

The effects of CLZ and JNJ at concentrations of 1, 10 and 25 µM on the cell viability of human neuroblastoma cell line SK-N-SH after 24 and 48 h are compared in Figure 1A. Two-way ANOVA (24 h: F_6,14_ = 90.12, *p* = 2.2 × 10^−10^; 48 h: F_6,14_ = 382.45, *p* = 1.1 × 10^−14^) showed a significant effect of drug treatment, concentration and their interaction. In Bonferroni post hoc pair comparisons, except for the lowest concentration, the CLZ treatment resulted in a significant decrease in cell viability, showing a concentration-dependent relationship after 24 h (by 12%: *p* = 3 × 10^−5^ and 26%: *p* = 3 × 10^−9^ at 10 and 25 µM, respectively) and after 48 h (by 5%: *p* = 0.008; 15%: *p* = 2 × 10^−8^ and 37%: 1 × 10^−13^ at 1, 10 and 25 µM, respectively). However, only a modest increase (6%: *p* = 6 × 10^−4^) and decrease (4%: *p* = 0.025) in cell viability was observed after 48 h of JNJ treatment at 10 and 25 µM, respectively.

After 24 h and 48 h, the viability of cells exposed to DA (100 µM) was reduced by 20% and 30%, respectively. To evaluate the capacity of CLZ or JNJ to reduce or increase the neurotoxicity induced in the cultures, cells exposed to DA alone were used as controls for the analysis. Two-way ANOVA (24 h: F_6,14_ = 77.29, *p* = 6.3 × 10^−10^; 48 h: F_6,14_ = 60.59, *p* = 3.2 × 10^−9^) also showed a significant effect of drug treatment, concentration and their interaction. Bonferroni post hoc pair comparisons showed that CLZ at concentrations of 10 and 25 µM enhanced this DA toxicity, as indicated by a concentration-dependent decrease in cell viability after 24 h (20%: *p* = 3 × 10^−6^ and 39%: *p* = 6 × 10^−10^, respectively) and 48 h (17%: *p* = 8 × 10^−4^ and 45%: *p* = 9 × 10^−9^, respectively) compared with cells treated with DA alone. To a lesser extent, cotreatment with the highest concentration of JNJ (25 µM) also enhanced DA toxicity, producing a decrease in cell viability of 17% at both exposure times (24 h: *p* = 2 × 10^−5^ and 48 h: *p* = 0.001). A decrease of 9% was also detected 24 h after JNJ cotreatment at 10 µM (*p* = 0.014) (Figure 1B). Therefore, with the same drug concentrations, cell cultures exposed to DA showed significantly higher cell viability when treated in combination with JNJ than when treated in combination with CLZ after 24 h (10 µM: 90% vs. 80%: *p* = 0.002; 25 µM: 80% vs. 60%: *p* = 6 × 10^−6^) and 48 h (10 µM: 94% vs. 82%: *p* = 0.023; 25 µM: 83% vs. 55%: *p* = 4 × 10^−6^).

Similar to the effects of DA exposure, the viability of cells exposed to GLU (80 mM) was reduced by 20% and 30% after 24 h and 48 h, respectively. To evaluate the capacity of CLZ or JNJ to reduce or increase the neurotoxicity induced in the cultures, cells exposed to GLU alone were used as controls for the analysis. Two-way ANOVA (24 h: F_6,14_ = 65.26, *p* = 2.0 × 10^−9^; 48 h: F_6,14_ = 102.04, *p* = 9.7 × 10^−11^) also showed a significant effect of drug treatment, concentration and their interaction. Again, cotreatment with CLZ enhanced GLU toxicity, as shown by a concentration-dependent decrease in cell viability after 24 h (9%: *p* = 0.008; 17%: *p* = 7 × 10^−6^; and 31 %: *p* = 2 × 10^−9^ at 1, 10 and 25 µM, respectively) and 48 h (17%: *p* = 1 × 10^−4^ and 38%: *p* = 6 × 10^−10^ at 10 and 25 µM, respectively) compared with cells exposed to GLU alone. Although slight differences were detected, the viability of cells cotreated with increasing concentrations of JNJ was similar to that of cells exposed only to GLU (Figure 1C).

### 2.2. Effects of Clozapine (CLZ) and JNJ-46356479 (JNJ) Alone and in Combination with Dopamine (DA) or Glutamate (GLU) on Caspase 3 Activity

**Figure 2:**
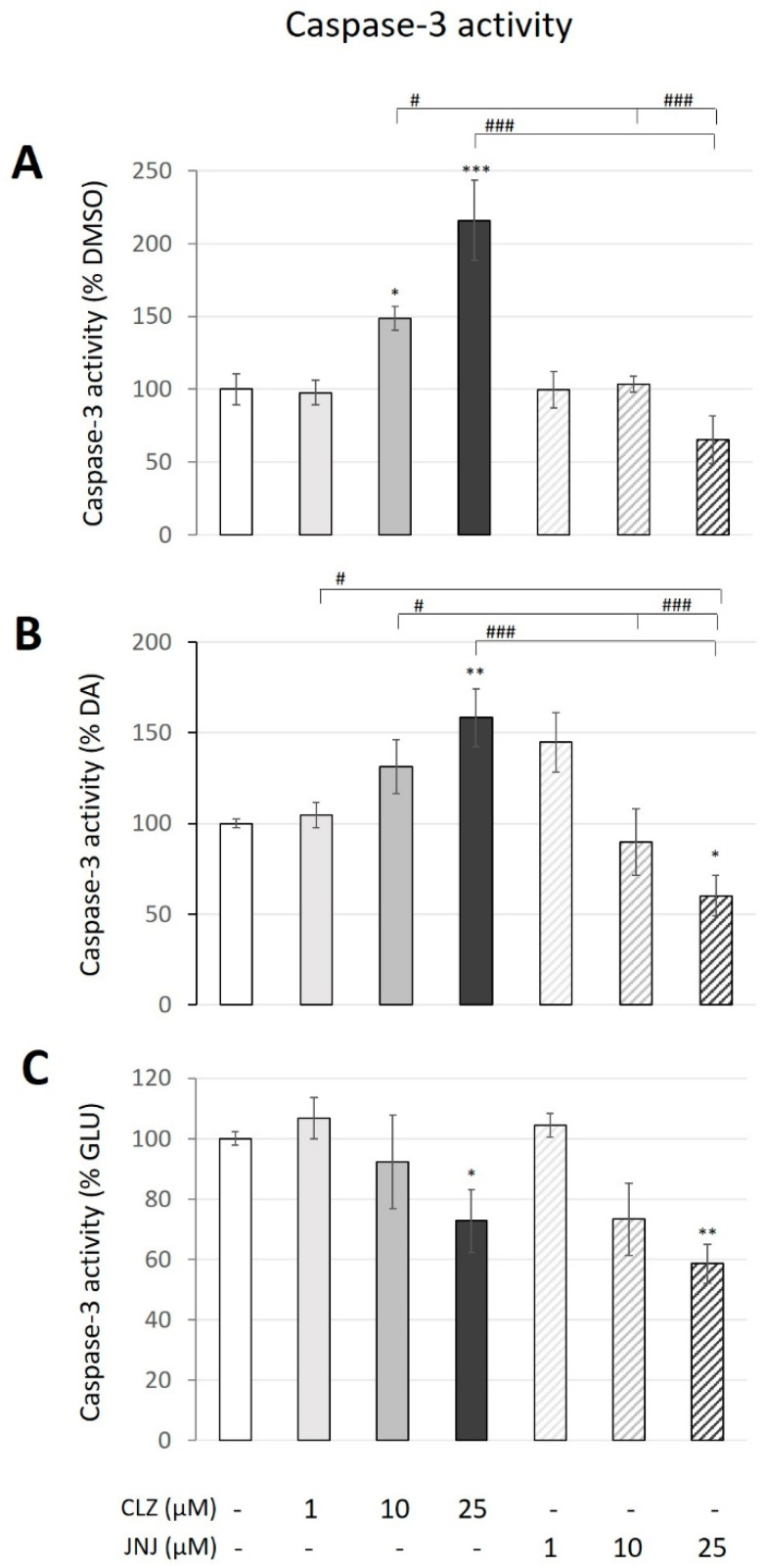
Effects of different concentrations (1, 10 and 25 μM) of clozapine (CLZ) and JNJ-46356479 (JNJ) alone (**A**), in combination with dopamine (DA) at 200 μM (**B**) and with glutamate (GLU) at 160 mM (**C**) on SK-N-SH caspase 3 activity after 24 h as measured by fluorometric assays. The values represent percentages of the control (DMSO-treated), DA-treated or GLU-treated cells and are expressed as means ± S.E.M (n = 3). Two-way ANOVA was performed to assess the drug treatment and concentration effect, and Bonferroni’s post hoc test was used for pair comparisons. * *p* ≤ 0.05, ** *p* ≤ 0.01, *** *p* ≤ 0.001 vs. control. ^#^ *p* ≤ 0.05, ^###^ *p* ≤ 0.001 included in square brackets indicate comparisons between CLZ and JNJ.

The effects of CLZ and JNJ at concentrations of 1, 10 and 25 µM on the caspase 3 activity, an apoptotic marker, in the SK-N-SH cell line after 24 h are compared in Figure 2A. Two-way ANOVA (F_6,14_ = 11.71, *p* = 9.0 × 10^−6^) showed a significant effect of drug treatment, concentration and their interaction. Bonferroni post hoc pair comparisons showed that CLZ significantly increased caspase 3 activity in a concentration-dependent manner (by 49%: *p* = 0.02 and by 116%: *p* = 0.0002 at 10 and 25 µM, respectively). The JNJ treatment did not affect caspase 3 activity.

In order to induce similar marked increases in apoptosis, the cells were exposed to high concentrations of both DA (200 µM) and GLU (160 mM), which produced a fivefold increase in caspase 3 activity under both conditions. To evaluate the capacity of CLZ or JNJ to reduce or increase the neurotoxicity induced in the cultures, cells exposed to DA or GLU alone were used as controls for the analysis. For DA exposure, two-way ANOVA (F_6,14_ = 6.41, *p* = 6.0 × 10^−4^) showed a significant effect of drug treatment and its interaction with concentration. Cotreatment with CLZ seemed to enhance the apoptotic effect of DA by producing concentration-dependent increases in caspase 3 activity, with a significant increase (of almost 60%) at the highest concentration (*p* = 0.003) (Figure 2B). In contrast, cotreatment with the highest JNJ concentration reduced the apoptotic effect of DA, decreasing caspase 3 activity by 40% compared to cells treated with DA alone (*p* = 0.03). For GLU exposure, two-way ANOVA (F_6,14_ = 3.77, *p* = 0.012) only showed a significant effect of concentration, as both CLZ and JNJ treatments showed a similar effect. Cotreatments with the highest concentration of both CLZ and JNJ also seemed to protect the cell cultures from GLU-induced apoptosis, as reductions in caspase 3 activity were observed compared to cells exposed only to GLU (by 27%: *p* = 0.045 and 41%: *p* = 0.007, respectively) (Figure 2C). Although JNJ cotreatment produced a partial normalization of this apoptotic parameter increased by DA and GLU, the significant reductions in caspase 3 activity did not reach the vehicle-treated cell values.

### 2.3. Effects of Clozapine (CLZ) and JNJ-46356479 (JNJ) Alone and in Combination with Dopamine (DA) or Glutamate (GLU) on Viable and Apoptotic Cells

**Figure 3:**
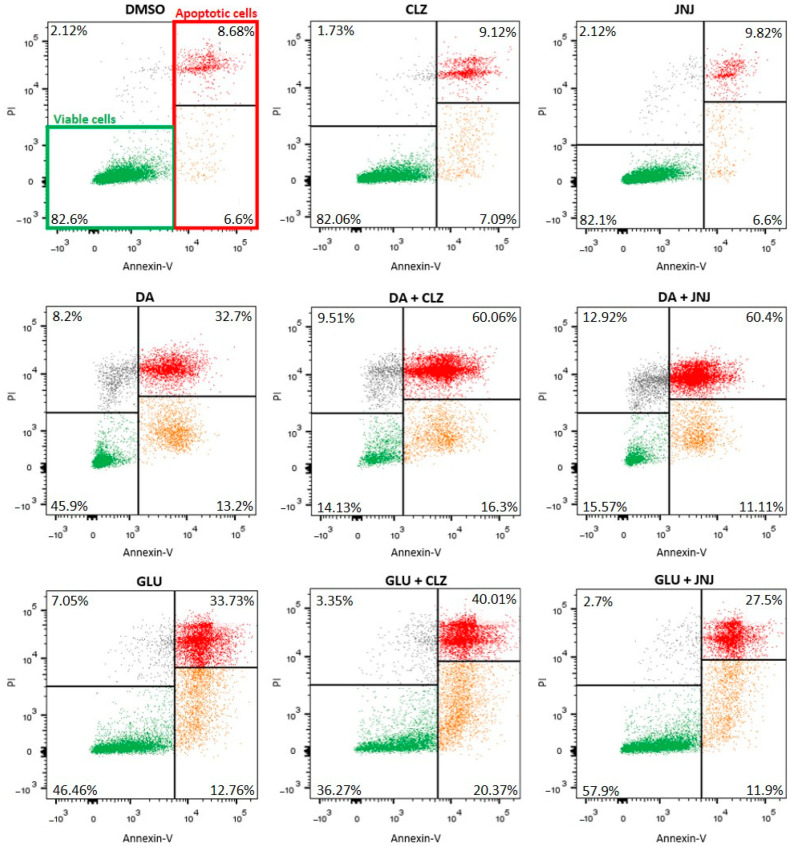
Representative plots showing apoptotic cell death as assessed by cytometric analysis using annexin-V/propidium iodide (PI). Dot plots represent typical results after SK-N-SH incubation with clozapine (CLZ) and JNJ-46356479 (JNJ) at the highest concentrations (25 μM) alone or in combination with dopamine (DA) at 200 μM or glutamate (GLU) at 160 mM. The fluorescence intensity from PI-stained cells is indicated on the *y*-axis, and the fluorescence intensity from cell-bound annexin-V-FITC is indicated on the *x*-axis. Live cells were negative for both annexin-V and PI (annexin-V–/PI–) (green cell population in the lower-left quadrant indicated by a green gate in the first representative plot), and apoptotic cells were positive for annexin-V (lower- and upper-right regions indicated by a red gate in the first representative plot). Among the dead cell population, cells positive for annexin-V and negative for PI (annexin-V+/PI−) were undergoing the early stages of apoptosis, in which the plasma membrane is still intact, and exclude PI (orange population in the lower-right quadrant). In the late stages of apoptosis and necrosis, dying cells can no longer exclude PI, and the upper-right region displays both annexin-V-positive and PI-positive (annexin-V+/PI+) cells (red cell population). Annexin-V-negative and PI-positive (annexin-V−/PI+) cells in the upper-left region are necrotic cells (grey population).

We used a flow cytometric assay to measure the externalization of phosphatidylserine using annexin-V/propidium iodide (PI). Representative plots are shown in Figure 3; live cells were found to be negative for both annexin-V and PI (annexin-V–/PI–) (lower left quadrant), and apoptotic cells were found to be positive for annexin-V (lower- and upper-right regions). Cells positive for annexin-V and negative for PI (annexin-V+/PI−) were undergoing the early stages of apoptosis, in which the plasma membrane is still intact, and exclude PI (lower-right quadrant). In the late stages of apoptosis and necrosis, dying cells can no longer exclude PI, and the upper-right region displays both annexin-V-positive and PI-positive (annexin-V+/PI+) cells. Annexin-V-negative and PI-positive (annexin-V−/PI+) cells in the upper-left region are necrotic cells.

**Figure 4:**
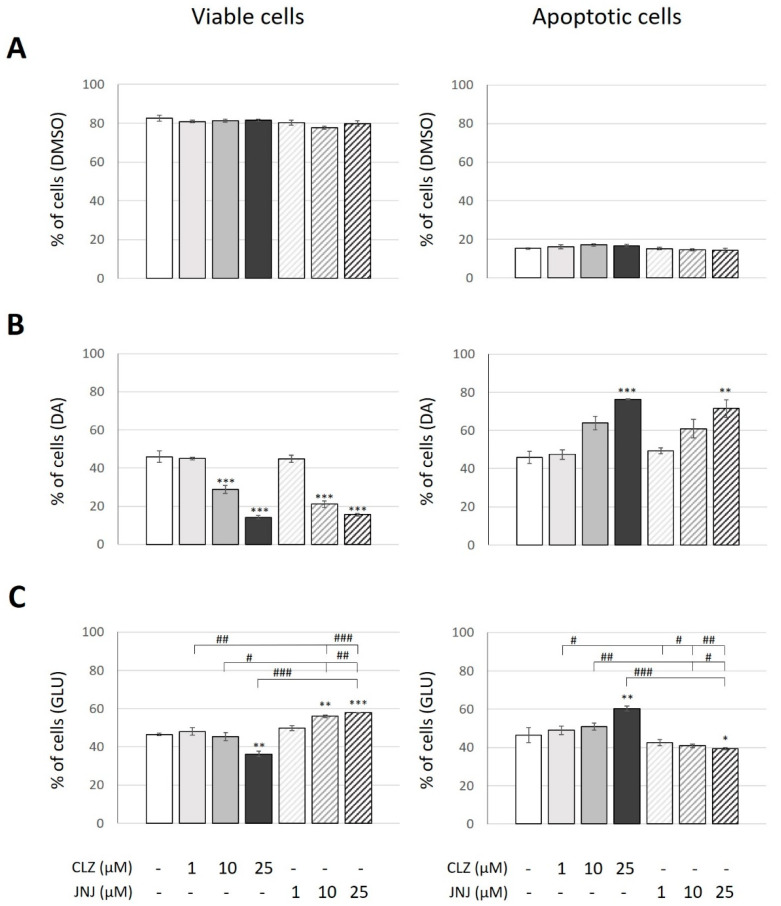
Effects of different concentrations (1, 10 and 25 μM) of clozapine (CLZ) and JNJ-46356479 (JNJ) alone (**A**), in combination with dopamine (DA) at 200 μM (**B**) or in combination with glutamate (GLU) at 160 mM (**C**) on SK-N-SH viability and apoptotic cell death after 24 h as assessed by flow cytometric analysis using annexin-V/propidium iodide (PI). Viable cells were counted as those negative for both annexin-V and PI (annexin-V–/PI–), and apoptotic cells were counted as those positive for annexin-V (annexin-V+/PI− plus annexin-V+/PI+). The values represent percentages of cells and are expressed as means ± S.E.M (n = 3). Two-way ANOVA was performed to assess the drug treatment and concentration effect, and Bonferroni’s post hoc test was used for pair comparisons. * *p* ≤ 0.05, ** *p* ≤ 0.01, *** *p* ≤ 0.001 vs. control. ^#^ *p* ≤ 0.05, ^##^ *p* ≤ 0.01, ^###^ *p* ≤ 0.001 included in square brackets indicate comparisons between CLZ and JNJ.

The effect of CLZ and JNJ at concentrations of 1, 10 and 25 µM on viable and apoptotic cell death (annexin-V+ cells) of the SK-N-SH cell line after 24 h was also assessed. The percentages of viable and apoptotic cells under the vehicle-treated condition were 83% and 15%, respectively. Cells treated with CLZ or JNJ also showed similar percentages of both viable and apoptotic cells, ranging from 78% to 85% and from 13% to 18%, respectively. No significance differences between experimental conditions were observed (Figure 4A).

Again, cell cultures were exposed to high concentrations of DA (200 µM) and GLU (160 mM) to induce similar marked increases in apoptotic cell death. Specifically, both inductions produced a threefold increase in the number of cells with externalized phosphatidylserine (46% of total cells) and showed about 50% reduction in live cell number (46% of total cells). To evaluate the capacity of CLZ or JNJ to reduce or increase the neurotoxicity induced in the cultures, cells exposed to DA or GLU alone were used as controls for the analysis. Two-way ANOVA (viable cells: F_6,14_ = 82.79, *p* = 1.3 × 10^−9^; apoptotic cells: F_6,14_ = 12.94, *p* = 8.1 × 10^−5^) only showed a significant effect of concentration for both viable and apoptotic cells and not for the type of drug treatment, as similar effects on viability and apoptotic cell death were observed when the cultures exposed to DA were cotreated with CLZ or JNJ. Both drugs enhanced the toxicity of DA in a concentration-dependent manner. Compared to cells exposed to DA alone, cotreatments with CLZ or JNJ showed reduced cell viability when medium and high concentrations were used (viable cells ranging from 15% to 29% of total cells); a reduction of at least 40% and 60%, respectively, was observed (CLZ at 10 µM and 25 µM: *p* = 0.0002 and *p* = 1.3 × 10^−7^; JNJ at 10 µM and 25 µM: *p* = 3 × 10^−6^ and *p* = 2.4 × 10^−7^, respectively). Accordingly, compared to cells only exposed to DA, cotreatments with high concentrations (25 µM) of CLZ or JNJ resulted in increased apoptosis, showing 76% and 71% apoptotic cells, respectively (CLZ: *p* = 0.001 and JNJ: *p* = 0.005) (Figure 4B).

However, opposite effects were observed on viability and apoptotic cell death when the cultures exposed to GLU were cotreated with CLZ or JNJ (Figure 4C). Two-way ANOVA (viable cells: F_6,14_ = 27.13, *p* = 6.1 × 10^−7^; apoptotic cells: F_6,14_ = 12.73, *p* = 5.8 × 10^−5^) showed a significant effect of drug treatment and its interaction with dosage. Whereas the CLZ treatment enhanced the toxicity of GLU, decreasing viable cells by 20% and increasing apoptotic cells by 30% compared to cultures exposed to GLU (about 36% and 60% of cells were viable or apoptotic cells, respectively) (25 µM: *p* = 0.003 and *p* = 0.006, respectively), JNJ seemed to protect against it by increasing the number of viable cells (by 21%: *p* = 0.005 and 25%: *p* = 0.001 at 10 and 25 µM, respectively) and decreasing apoptotic cell death by about 15% (25 µM: *p* = 0.027) (about 57% and 40% of cells were viable or apoptotic cells, respectively). Although cotreatment with JNJ did not reach the vehicle-treated cell values, it again produced a partial normalization of an apoptotic parameter induced by GLU.

### 2.4. Effects of Clozapine (CLZ) and JNJ-46356479 (JNJ) Alone and in Combination with Dopamine (DA) or Glutamate (GLU) on Gene Expression

**Figure 5:**
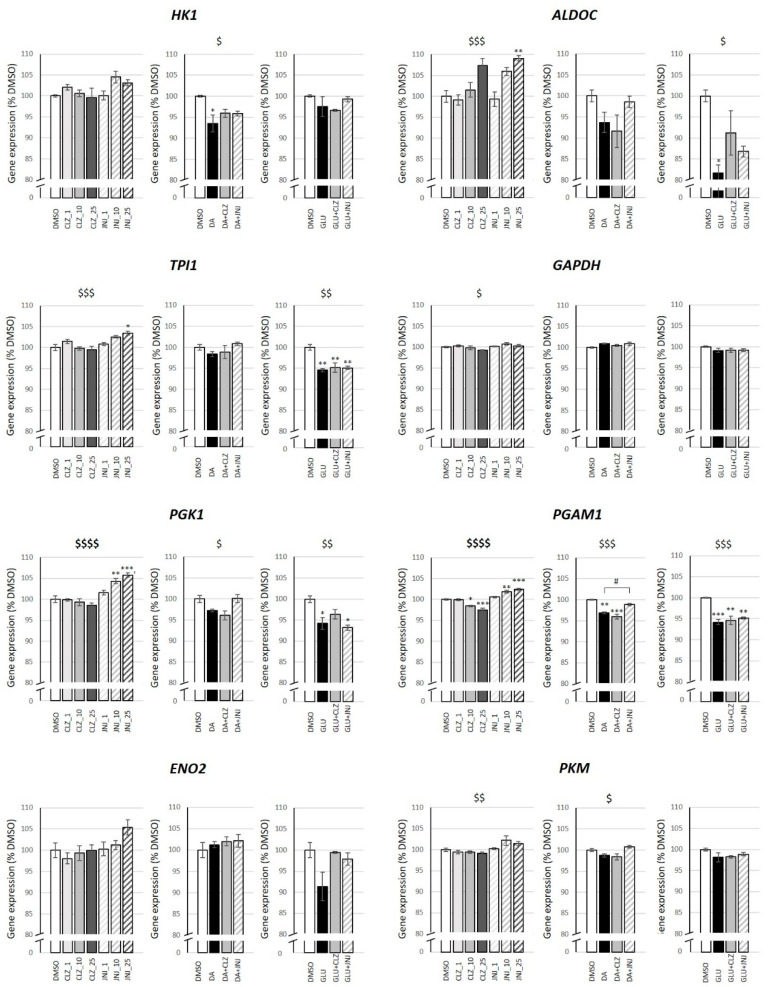
Effects of different concentrations of clozapine (CLZ) and JNJ-46356479 (JNJ) alone and in combination with dopamine (DA) at 100 μM or in combination glutamate (GLU) at 80 mM on the expression levels of glycolytic genes. Values represent percentages of the control (DMSO-treated cells) and are expressed as means ± S.E.M (n = 3). Two-way ANOVA was performed to assess the drug treatment and concentration effect on cells treated with CLZ (1, 10 and 25 μM) or JNJ (1, 10 and 25 μM) alone. One-way ANOVA was performed to assess the drug treatment effect on cells exposed to DA or GLU alone or in combination with CLZ (10 μM) or JNJ (10 μM). Significant associations for ANOVAs are shown as ^$^ *p* ≤ 0.05, ^$$^ *p* ≤ 0.01, ^$$$^ *p* ≤ 0.001 and **^$$$$^** *p* < 7.3 × 10^−5^ (Bonferroni corrected value: 0.05/226 genes × 3 experimental conditions). Bonferroni’s post hoc test was used for pair comparisons. * *p* ≤ 0.05, ** *p* ≤ 0.01, *** *p* ≤ 0.001 vs. control; ^#^ *p* ≤ 0.05 vs. DA. *ADCY5*, adenylate cyclase 5; *ALDOC*, aldolase fructose-bisphosphate C; *ENO2*, enolase 2; *GAPDH*, glyceraldehyde-3-phosphate dehydrogenase; *HK1*, hexokinase 1; *JUN*, Jun proto-oncogene; *PGAM1*, phosphoglycerate mutase 1; *PGK1*, phosphoglycerate kinase 1; *PKM*, pyruvate kinase M1/2; *TPI1*, triosephosphate isomerase 1.

We analyzed the expression changes in a total of 226 key genes involved in survival and cell death that could be triggered by DA and GLU and also related to the CLZ and JNJ mechanisms of action [30,31] (Supplementary Table S1). After Bonferroni correction (*p* < 7.3 × 10^−5^ (0.05/226 genes × 3 experimental conditions)), significant changes in gene expression were observed for jun proto-oncogene (*JUN*) (F_6,14_ = 22.34, *p* = 2.0 × 10^−6^), adenylate cyclase 5 (*ADCY5*) (F_6,14_ = 15.30, *p* = 2.0 × 10^−5^), phosphoglycerate kinase 1 (*PGK1*) (F_6,14_ = 19.95, *p* = 4.0 × 10^−6^) and phosphoglycerate mutase 1 (*PGAM1*) (F_6,14_ = 42.49, *p* = 3.4 × 10^−8^) genes in cells treated with CLZ or JNJ alone. Particularly, as shown in the Bonferroni post hoc analysis, CLZ treatment significantly reduced the gene expression of *JUN* (10 µM: *p* = 0.017; 25 µM: *p* = 4.2 × 10^−5^), *ACYD5* (25 µM: *p* = 4.2 × 10^−5^) and *PGAM1* (10 µM: *p* = 0.026; 25 µM: *p* = 0.00024), and JNJ increased gene expression of *PGK1* (10 µM: *p* = 0.0036; 25 µM: *p* = 0.00018) and *PGAM1* (10 µM: *p* = 0.0036; 25 µM: *p* = 0.0005). Given that both *PGK1* and *PGAM1* codify for glycolytic enzymes, we focused on the rest of the glycolysis pathway genes. Interestingly, although significance did not survive Bonferroni correction, differential expression was detected at a nominal level (*p* < 0.05) in the majority of these genes when cells were treated with CLZ or JNJ alone, as well as in those exposed to DA and GLU, including hexokinase 1 (*HK1*) (DA: F_3,8_ = 5.25, *p* = 0.027), aldolase fructose-bisphosphate C (*ALDOC*) (F_6,14_ = 8.692, *p* = 4.5 × 10^−4^; GLU: F_3,8_ = 6.89, *p* = 0.013), triosephosphate isomerase 1(*TPI1*) (F_6,14_ = 7.57, *p* = 9.1 × 10^−4^; GLU: F_3,8_ = 12.71, *p* = 0.002), glyceraldehyde-3-phosphate dehydrogenase (*GAPDH*) (F_6,14_ = 2.91, *p* = 0.047), *PGK1* (F_6,14_ = 19.95, *p* = 4.0 × 10^−6^; DA: F_3,8_ = 6.05, *p* = 0.019; GLU: F_3,8_ = 8.50, *p* = 0.007), *PGAM1* (F_6,14_ = 42.49; *p* = 3.4 × 10^−8^; DA: F_3,8_ = 26.71, *p* = 1.6 × 10^−4^; GLU: F_3,8_ = 20.66, *p* = 4.0 × 10^−4^) and pyruvate kinase M1/2 (*PKM*) (F_6,14_ = 4.93, *p* = 0.006; DA: F_3,8_ = 5.56, *p* = 0.023). Despite the observed significance, it should be noted that the percentages of gene expression change were subtle in many cases. No differences were detected for the enolase 2 (*ENO2*) gene. In general, the expression of the majority of these genes was increased with JNJ treatment. Moreover, DA and GLU exposure decreased expression of these genes, with significant differences in post hoc analysis for *HK1*, *ALDOC*, *TPI1*, *PGK1* and *PGAM1*, which were then partially normalized in many cases when cells were cotreated with JNJ (Figure 5).

## 3. Discussion

The results of this study provide evidence of the drug safety of JNJ in neuroblastoma cell cultures exposed to increasing concentrations of this molecule and of its potential neuroprotective effect against apoptosis and cell death, particularly decreasing the caspase 3 activation induced by DA and GLU and increasing and decreasing the number of viable and apoptotic cells, respectively, when cultures were exposed to GLU. Moreover, JNJ treatment showed enhanced safety and neuroprotective effects compared with CLZ treatment at the cellular level. The capacity of JNJ to partially normalize altered expression of glycolytic genes could act as a protective factor and be related to its presumed neuroprotective effect observed in our cell cultures.

In the last decade, intensive research has been focused on the development of the mGluR2 PAM to treat neuropsychiatric disorders such as schizophrenia, particularly its negative and cognitive symptoms. Although schizophrenia clinical trials returned negative results with previous molecules, such as JNJ-40411813 or AZD8529, further preclinical and clinical studies may contribute to defining the potential of these and other improved mGluR2 PAMs as novel therapeutic agents, as well as their optimal therapeutic uses [32]. In this study, we evaluated the in vitro neuroprotective activity of JNJ, a new mGluR2 PAM with a more balanced profile and improved drug-like attributes than previously reported leads [18]. In a recent in vivo study performed by our group, this current lead was found to partially improve neuropathological deficits and schizophrenia-like behavior in a postnatal ketamine mouse model [20].

In the present study, JNJ at different concentrations and treatment times did not affect cell viability; only slight changes were observed when cells were treated with the highest concentration for 48 h. Moreover, this pharmacological treatment did not induce apoptosis in the cell cultures, as caspase 3 activity and phosphatidylserine externalization were not affected. This is in accordance with the lack of toxicity observed in neuronal cultures treated with similar concentrations of another mGluR2 PAM [33]. These results indicate that JNJ is not neurotoxic and could prove to have good tolerability in clinical practice, as previously observed with JNJ-40411813 [34]. Indeed, the drug safety of JNJ seems to be superior to that of CLZ, which produced concentration-dependent decreases in cell viability and increases in caspase 3 activity. In vitro cytotoxic effects of CLZ have also been reported. There is other evidence of CLZ treatment resulting in similar decreases in the viability of different types of cell cultures, including human neuroblastoma [35,36]. Lundeberg et al. [37] detected cytotoxic activity when primary mouse neural stem cells were exposed to ≥500 nM of CLZ, and a study by Park et al. [38] also showed neurotoxic activity when rat primary neurons were exposed to ≥10 μM of CLZ in vitro, which was associated with increased apoptosis and autophagy.

However, there is considerable evidence of the neuroprotective effects of second-generation APs [21,39]. Lundberg and colleagues also reported that CLZ may have a protective/antiapoptotic effect on neural stem cells when used at lower concentrations (10 nM), supporting their previous in vivo observations indicating that CLZ promotes neurogenesis [37]. This is in accordance with results obtained by Takaki et al. [40], which revealed an increased number of spines in cultured cortical rat neurons when they were treated with CLZ at 1 µM but a decreased in their number when they were treated with high concentrations of CLZ (10 µM). This indicates that the dose selection of APs may be important for the protection of dendritic spines in patients with schizophrenia [40]. Similarly, CLZ at 1 µM increased spine density in rat hippocampal neurons [41]. Our results are in line with this finding, as low CLZ concentrations did not affect neuroblastoma viability, apoptosis or cell death, but high CLZ concentrations were toxic and induced cell death.

It is important to note that most evidence of the neuroprotective effects of APs has been observed when cell cultures were exposed to different types of cell damage or stress [21,36,42]. In this study, we induced neurotoxicity with high concentrations of DA and GLU as reported in other studies with neuroblastoma cell lines [21,43,44,45,46]. GLU and DA are essential neurotransmitters, but in excess in the extracellular environment, they may induce neuronal damage and degeneration, which is involved in the pathophysiology of schizophrenia. Different mechanisms are involved in such neurotoxicity, including oxidative stress, oxytosis, mitochondrial damage and cellular apoptosis [13,43,45,46], which could be affected in different ways by CLZ and JNJ treatments. JNJ showed a potential neuroprotective effect in vitro, as it reduced the activation of caspase 3 induced by DA. However, we observed an increase in apoptotic cells when cultures exposed to DA were cotreated with JNJ, probably due to caspase 3-independent cell death processes [21]. Again, compared with JNJ, CLZ showed more potent cytotoxic effects, as it induced a greater enhancement of DA toxicity, producing greater decreases in cell viability and greater increases in caspase 3 activity. These differences could be due to the capacity of JNJ to prevent the caspase 3 activation induced by DA that we observed in our cultures. Along the same lines, when neurotoxicity was induced by GLU exposure, the JNJ treatment again showed a relative neuroprotective effect, decreasing the activation of caspase 3. Unlike in cells exposed to DA, in the GLU model, this effect was observed in parallel with an increase in cell viability and a reduction in apoptotic cell death, as determined by cytometric analysis. These discrepancies in JNJ effects in each model of cell damage could be explained by the induction of cell death by the different neurotoxic insults through distinct mechanisms, which could be differently modulated by the drug. Once more, JNJ was less neurotoxic than CLZ, which enhanced both the decrease in cell viability and the increase in apoptotic cell death induced by GLU, despite the fact that the highest concentration of CLZ also showed an apparent neuroprotective effect against the activation of caspase 3 induced by GLU. Cell exposure to high concentrations of GLU can lead to different cell death processes including oxytosis [13]. Given that a different neurotoxic/neuroprotective profile between CLZ and JNJ was observed in our cell model, these drugs may exert their action through different cell death mechanisms. Future studies measuring specific markers of apoptosis, oxytosis or necroptosis may clarify this issue. Other authors have shown a neuroprotective effect of mGluR2/3 agonists against different cytotoxic molecules in neuronal cells, including undifferentiated and differentiated neuroblastoma cells, by avoiding decreases in cell viability. However, Jantas et al. [23] reported that this neuroprotection effect was independent of caspase 3 activity. Differences in the mechanism of action of the mGluR2/3 modulators, cell culture type used and death pathways activated by different neurotoxic insults could explain these discrepancies.

In order to explore molecular pathways that could be related to the neurotoxic/neuroprotective effects of CLZ and JNJ observed in vitro, we analyzed changes in expression of key genes involved in survival and cell death that could be triggered by DA and GLU, such as apoptosis, necroptosis or oxytosis [43,44,45,46]. Interestingly, although the percentages of gene expression change were subtle in many cases, JNJ increased the expression of most glycolytic genes, especially that of *PGK1* and *PGAM1*, and was capable of normalizing their expression when reduced by DA and GLU exposures. There are multiple pieces of evidence for decreased activity of major glycolytic enzymes in schizophrenia [47]. In this scenario, neurons would not be able to adequately meet their energetic demands via glycolysis nor through oxidative phosphorylation, which could induce apoptosis. In fact, glycolytic enzymes have been proposed as apoptotic biomarkers [48]. Proteomics in the prefrontal cortex and cerebrospinal fluid of patients with schizophrenia have shown altered levels of glycolytic enzymes, including a reduction in *PGAM1* expression [49,50], which was also observed in a phencyclidine animal model of schizophrenia [51]. Similar gene expression alterations were found in our cellular model and were partially reverted to normal expression levels with JNJ, which could act as a protective factor and be related to its putative neuroprotective effect observed in SK-N-SH cells. This effect could be mediated by mGluR2, given the high affinity of JNJ for this receptor, although the drug interaction with other targets, such as mGluR3 and other mGluRs [18], could also be responsible for this effect. It should be noted that *GRM2*, *GRM7* and *GRM8* are the mGluR genes that showed the highest expression values in our SK-N-SH cells, which is in accordance with data reported in SH-SY5Y neuroblastoma cells [23].

One of the pathophysiological theories of schizophrenia and its negative and cognitive symptomatology is the existence of increased synaptic pruning during crucial stages of brain development, such as adolescence, which could be due to increased neuronal apoptosis resulting from excessive glutamatergic neurotransmission [8,52]. First-episode schizophrenia patients seem to have increased basal apoptotic mechanisms and to be more vulnerable to a proapoptotic environment [10]. It has therefore been suggested that future drugs should focus on aberrant neurodevelopment and disease progression [53]. One such approach would be to reduce the GLU storm and the excess of dendritic apoptosis [8]. The pharmacological effects of mGluR2 PAMs such as JNJ in reducing the presynaptic release of GLU and possibly preventing caspase 3 activation in both hyperdopaminergic and hyperglutamatergic states, as observed in this study, could be considered one of the main future pharmacological strategies. The use of this kind of GLU modulator in critical periods such as prodromes or the transition to psychosis when there could be a neurotoxic storm of GLU [54] could be key in preventing the onset of negative and cognitive symptoms or in improving their control [8,41].

In the present study, we selected a common range of drug concentrations based on our previous experience and other reported studies that assessed CLZ and different mGluR2/3 agonists or modulators in cell cultures [22,23,24,29,35,38,55]. It should be noted that although there are no clinical data for JNJ, the doses of JNJ-40411813 used in the exploratory phase 2a clinical trial were up to 150 mg/day [32], so they correspond to low clinical doses of CLZ. Nevertheless, JNJ seems to be less neurotoxic and more neuroprotective than the same or even lower doses of CLZ.

The neuroblastoma cell line displays neuronal properties and is a popular in vitro model used in neuropsychiatric research including schizophrenia [25,26]. It has the advantage of low cost, ease of culture, reproducibility and available literature, as it has been widely used to test pharmacological effects of drugs including APs [27,28,29]. However, it should be noted that working with this cell model has its limitations. It is difficult to obtain therapeutically meaningful results, as the effects induced by the different treatments evaluated in vitro could vary at the brain level of patients with schizophrenia. Moreover, as previously discussed, changes in concentrations of drugs may induce different effects on cell cultures. The use of only one type of cell model is also a limitation of the present study. Different results could be observed by using differentiated neuroblastoma [23,24] or other types of neuronal cells. The analysis of other apoptotic markers such as DNA damage would be also interesting. Another limitation is that changes in gene expression were only assessed by microarray analysis. Future studies with Western blot or qPCR are necessary to confirm our results, as well as functional analysis of the identified glycolytic genes. Moreover, different drug, DA and GLU concentrations could be used to assess their differential effects on gene expression. Translational research using cell, animal and human models is needed in order to deepen our knowledge of the mechanisms of action of APs. Although our results should be considered preliminary and need to be replicated in different types of neuronal cells, this in vitro study provides new evidence concerning the neurotoxic/neuroprotective mechanisms of CLZ and especially of the mGluR2 PAM JNJ-46356479.

## 4. Material and Methods

### 4.1. Reagents

Minimum essential medium (MEM), fetal bovine serum (FBS), L-glutamine, sodium pyruvate, penicillin, streptomycin, phosphate buffered saline (PBS) and trypsin were purchased from Life Technologies (Carlsbad, CA, USA). DA and dimethyl sulfoxide (DMSO) were obtained from Sigma-Aldrich (St. Louis, MO, USA). CLZ was purchased from Tocris Bioscience (Bristol, UK). Janssen-Cilag S.A. provided the compound JNJ-46356479 through a material transfer agreement.

### 4.2. Cell Culture

SK-N-SH human neuroblastoma cells were purchased from the American Type Cell Culture (ATCC, Manassas, VA, USA) and were cultured in MEM supplemented with 10% FBS, 2 mM L-glutamine, 500 µM sodium pyruvate, 50 units/mL penicillin and 50 µg/mL streptomycin. The cells were grown in a humidified incubator with 5% CO_2_ at 37 °C. The culture medium was changed every 2–3 days. All experiments were performed within passages 6–12. Technical replicates were performed within the same passage number.

Based on our previous experience in assessing different APs in SK-N-SH cells [21] and previous studies testing CLZ in human neuroblastoma cell cultures [26,29,40] or primary murine neurons [32], a common range of CLZ and JNJ concentrations including 1, 10 and 25 µM were tested in our experiments [22,23,24,29,35,38,55].

Based on previous tests and other studies performed on neuroblastoma cell lines, apoptosis and cell death was induced with high concentrations of DA and GLU [21,43,44,45]. Specifically, DA at 100 µM and GLU at 80 mM were used to induce similar reductions in cell viability. Double these concentrations of DA (200 µM) and GLU (160 mM) were used to similarly induce marked increases in apoptosis and cell death.

### 4.3. Cell Viability

SK-N-SH cells were seeded on 24-well plates at a density of 150,000 cells/well and treated with CLZ or JNJ at concentrations of 1, 10 and 25 µM, either alone or in combination with DA 100 µM or GLU 80 mM. Controls were treated with vehicle (0.25% DMSO, *v/v*) either alone or in combination with DA or GLU. Each condition was assessed by three biological replicates with three technical replicates each. Cell viability was determined by use of Alamar Blue^®^ (Sigma, St. Louis, MO, USA). Resazurin, a non-fluorescent indicator dye, is converted to bright-red fluorescent resorufin via the reduction reactions of metabolically active cells. The amount of fluorescence produced is proportional to the number of living cells. After 24 h and 48 h of incubation, 50 µL of Alamar Blue^®^ was added to each well and incubated for 2 h. Fluorescence was measured at an excitation wavelength of 540 nm and an emission wavelength of 590 nm using a Tecan Spark^®^ 20M microplate fluorescence reader (Tecan, Männedorf, Switzerland). Each measurement was performed at least in duplicate. Cell viability is expressed as a percentage of the control (vehicle-treated), DA-treated or GLU-treated cells.

### 4.4. Caspase 3 Activity as an Apoptotic Marker

Cells were cultured at a density of 120,000 cells/well on 12-well plates until 70–80% confluence. Then, the normal culture medium was replaced by culture medium containing CLZ and JNJ at concentrations of 1, 10 and 25 µM, either alone or in combination with DA at 200 µM or GLU at 160 mM. The culture medium for the controls contained the vehicle (0.25% DMSO, *v/v*) either alone or in combination with DA or GLU. Each condition was assessed by three biological replicates with two technical replicates each. After 24 h of drug exposure, caspase 3 activity was measured by the cleavage of acetyl-Asp-Glu-Val-Asp (Ac-DEVD) peptide-conjugated 7-amino-4-methyl-coumarin (AMC) using a caspase 3 fluorometric assay kit (Sigma, St. Louis, MO, USA). Cell pellets were incubated with 20 µL of ice-cold cell lysis buffer on ice for 20 min. The lysates were centrifuged at 4 °C for 15 min at 14,000× *g*. A total of 6 µL of the supernatant was transferred to a 96-well plate; then, 200 µL of reaction buffer containing the caspase 3 substrate (DEVD-AMC) was added to each well. To verify that the signal detected by the reaction was due to protease activity, an induced sample was incubated at 37 °C with the caspase 3 inhibitor acetyl-Asp-Glu-Val-Asp-al (Ac-DEVD-CHO) before adding the substrate. The AMC fluorescence counts in the wells were measured with a 360 nm excitation filter and a 465 nm emission filter at least in duplicate using a Tecan Spark^®^ 20M microplate fluorescence reader (Tecan, Männedorf, Switzerland). The fluorescence of the blanks was subtracted from each value, and the resultant fluorescence values were converted to caspase 3 activity using a standard curve for AMC. Caspase 3 activity was normalized to the total protein content of the cell extracts as measured by a DC protein assay kit (BioRad, Hemel Hempstead, UK). Results are expressed as percentage of control (vehicle-treated), DA-treated or GLU-treated cells.

### 4.5. Flow Cytometry Measurement of Apoptotic Cell Death Using Annexin-V/PI

We used an annexin V-FITC (fluorescein isothiocyanate) cell membrane labeling assay to detect the translocation of phosphatidylserine from the inner face of the cell membrane to the outer surface as a marker of apoptosis and cell death. PI was used to label the DNA in the cells where the cell membrane had been compromised. The assay was performed using an annexin-V-Fluos staining kit (Roche Diagnostics, Penzberg, Germany).

Cells were cultured at a density of 120,000 cells/well on 12-well plates until 70–80% confluence. Then, the normal culture medium was replaced by culture medium containing CLZ and JNJ at concentrations of 1, 10 and 25 µM, either alone or in combination with DA at 200 µM or GLU at 160 mM. The culture medium for controls contained vehicle (0.25% DMSO, *v/v*) either alone or in combination with DA or GLU. Each condition was assessed by three biological replicates with two technical replicates each. After 24 h of drug exposure, the SK-N-SH cells were harvested by trypsinization, and any floating cells were added back to the trypsinized cells and pelleted by centrifugation at 300× *g* for 5 min. A total of 200,000 cells were resuspended twice in cold PBS and spun at 300× *g* for 5 min. The pellet was resuspended in 100 µL of annexin-V-Fluos labeling solution and incubated with 0.6 µL FITC-conjugated annexin-V and 1.2 µL of PI for 15 min at room temperature in the dark. The samples were kept on ice and analyzed on a BD FACSCANTO flow cytometer equipped with two lasers (BD Bioscience, San Jose, CA, USA). Emission fluorescence was measured with a 525/50 filter for FITC and with a 610/20 filter for red PI. FITC and PI were excited with two different lasers at 488 nm for the first and 561 nm for the second, avoiding signal compensation. Data were analyzed using FlowJo software version 10.1r5 (Ashland, OR, USA). A minimum of 10,000 events were collected for each sample. For each experimental situation, the quadrants were adjusted depending on the respective controls.

### 4.6. Gene Expression Analysis

Cells were cultured at a density of 120,000 cells/well on 12-well plates until 70–80% confluence. Then, the normal culture medium was replaced by culture medium containing CLZ and JNJ at concentrations of 1, 10 and 25 µM alone and at a concentration of 10 µM in combination with DA at 100 µM or GLU at 80 mM. The culture medium for the controls contained the vehicle (0.25% DMSO, *v/v*) either alone or in combination with DA or GLU. Each condition was assessed by three biological replicates with two technical replicates each. After 24 h of drug exposure, cells were harvested and homogenized in TRIzol reagent (Life Technologies, Foster City, CA, USA), cells from technical replicates were collected and RNA was isolated following the manufacturer’s instructions. RNA quantity and quality were determined using a Nanodrop ND-2000 spectrophotometer (NanoDrop, Wilmington, DE, USA), and the integrity of RNA was assessed using an Agilent 2100 Bioanalyzer (Agilent Technologies, Palo Alto, CA, USA). A total of 500 ng of purified RNA from each sample was submitted to the Genomics core facility of the Institut d’Investigacions Biomèdiques August Pi i Sunyer (IDIBAPS, Barcelona, Spain) for labeling and hybridization to the Clariom S Human Array (Affymetrix, Santa Clara, CA, USA) according to the manufacturer’s protocol. Gene expression data preprocessing was performed using the Oligo R package [56]. The data were standardized using robust multichip analysis. Multiple probes mapping to the same gene were merged using the average as the summary of the hybridization values.

We selected key genes involved in survival and cell death that could be triggered by DA and GLU and related to the mechanism of action of CLZ and JNJ. Despite the complexity, given the notable crosstalk between signaling pathways, we selected a total of 226 genes related to extrinsic and intrinsic apoptosis, necroptosis, oxytosis, oxidative stress, autophagy, mitochondrial dysfunction, deregulation of oxidative phosphorylation and energy failure (Supplementary Table S1).

### 4.7. Statistical Analysis

Data were analyzed using IBM SPSS statistics 20 (IBM Corp., Chicago, IL, USA). Data were normally distributed (Shapiro–Wilk test). Statistical analysis was performed using one-way or two-way ANOVA (treatment and concentration) as appropriate, and mean values for each experimental group were compared using Bonferroni’s post hoc tests. Two-tailed *p*-values < 0.05 were considered significant. To avoid false-positive results in the gene expression analysis, we applied the Bonferroni correction method for multiple testing (0.05/number of comparisons). Statistical significance was reached at *p* < 7.3 × 10^−5^ (0.05/226 genes × 3 experimental conditions). For genes with significant changes in expression, Bonferroni’s post hoc test was used to compare each pair of groups.

## 5. Conclusions

For the first time, the results of the present study demonstrate that JNJ-46356479, a recently developed mGluR2 PAM, is not neurotoxic and attenuates apoptosis in neuroblastoma cell cultures, particularly decreasing the caspase 3 activation induced by DA and GLU and increasing and decreasing the number of viable and apoptotic cells, respectively, only when cultures were exposed to GLU. Its effects seem to be less neurotoxic and more neuroprotective than those observed with CLZ treatments. More studies are needed to determine the mechanisms of action of this GLU modulator and its potential as a novel therapeutic agent for schizophrenia.

## Data Availability

The data presented in this manuscript are available from the corresponding author upon request.

## Supplementary Materials

The following supporting information can be downloaded at: https://www.mdpi.com/article/10.3390/ijms24032054/s1.
